# Serum Mitochondrial Open Reading Frame of the 12S rRNA-c (MOTS-c) Dynamics as a Complementary Marker of Treatment Response in Newly Diagnosed Multiple Myeloma: A Prospective Analysis

**DOI:** 10.7759/cureus.98204

**Published:** 2025-11-30

**Authors:** Veysel Erol, Esin Avci, Sibel Kabukcu Hacioglu, Nil Guler, Gulsum Akgun Cagliyan, Başak Ünver Koluman, Ismail Can Kendir, Ozde Elver, Kayihan Kara, Erhan Kaya, Nevin Alayvaz Aslan

**Affiliations:** 1 Department of Hematology, Kahramanmaras Necip Fazil City Hospital, Kahramanmaras, TUR; 2 Department of Biochemistry, Pamukkale University Hospital, Denizli, TUR; 3 Department of Hematology, Pamukkale University Hospital, Denizli, TUR; 4 Department of Hematology, Aydin State Hospital, Aydin, TUR; 5 Department of Public Health, Faculty of Medicine, Kahramanmaraş Sütçü İmam University, Adana, TUR

**Keywords:** mitochondrial peptide, molecular biomarker, mots-c, multiple myeloma prognosis, treatment response

## Abstract

Objectives: This study evaluated whether serum levels of mitochondrial open reading frame of the 12S rRNA-c (MOTS-c) could provide prognostic insights in patients newly diagnosed with multiple myeloma (MM), particularly in relation to therapeutic responsiveness.

Methods: In a prospective analysis involving 29 MM patients, serum MOTS-c concentrations were measured before and after frontline treatment. Associations between MOTS-c fluctuations and clinical-biochemical variables were systematically explored.

Results: Patients who responded to treatment exhibited a substantial post-therapy increase in MOTS-c levels, while refractory cases showed little to no change. Post-treatment MOTS-c values inversely correlated with calcium and neutrophil count changes. Despite limited standalone predictive capacity, MOTS-c dynamics may reflect microenvironmental and metabolic adaptation linked to treatment response.

Conclusions: Changes in serum MOTS-c concentrations following therapy may serve as complementary markers of clinical response in MM. Further validation in larger cohorts is warranted.

## Introduction

Multiple myeloma (MM) ranks as the second most frequently diagnosed hematologic malignancy, accounting for approximately 1% of all cancers and 10% of blood-related neoplasms [1]. The disease exhibits a slight male predominance and primarily affects individuals over the age of 65 [2]. Although its precise etiology remains undefined, environmental exposures are believed to play a critical role in pathogenesis. MM often evolves from precursor conditions such as monoclonal gammopathy of undetermined significance (MGUS) and smoldering multiple myeloma (SMM). The annual progression rate from MGUS to MM is estimated at 1% [3], whereas SMM shows a 10% transformation rate in the initial five years, decreasing to 3% in the subsequent five years, and about 1.5% annually thereafter [4]. The risk of progression from MGUS or SMM is influenced by the proportion of plasma cells in the bone marrow, serum M-protein levels, and genetic abnormalities [5].

Over the last 25 years, significant improvements in overall survival have been achieved in MM management, largely due to the incorporation of agents such as proteasome inhibitors (bortezomib, carfilzomib, ixazomib), immunomodulatory drugs (thalidomide, lenalidomide, pomalidomide), CD38 monoclonal antibodies (daratumumab, isatuximab), SLAM7-targeted therapies (elotuzumab), and bispecific B-cell maturation antigen (BCMA)-targeted agents (teclistamab, elranatamab) [6]. According to Howlander et al., these advancements have contributed to a 54% increase in five-year survival and an 18% reduction in mortality [7].

Study objectives

The primary objective of this study was to determine whether serum mitochondrial open reading frame of the 12S rRNA-c (MOTS-c) levels change in response to treatment in newly diagnosed multiple myeloma patients and whether these changes are associated with treatment response. The secondary objectives were: (i) to compare baseline and post-treatment MOTS-c concentrations between responders and refractory cases, (ii) to assess the discriminatory capacity of MOTS-c using ROC analysis, and (iii) to explore potential relationships between MOTS-c dynamics and biochemical parameters reflecting bone metabolism, inflammation, or disease burden. By clearly defining these endpoints, the study aimed to provide preliminary insight into the clinical and biological relevance of MOTS-c as a potential adjunctive biomarker in multiple myeloma.

Pathogenesis of MM

The bone marrow microenvironment (BMME) is fundamental to the pathophysiology of MM. Reactive oxygen and nitrogen species, generated in response to hypoxia and inflammatory stimuli, activate cellular components of the BMME, including neutrophils, macrophages, dendritic cells, regulatory T cells, and adipocytes. This activation triggers the release of various cytokines, chemokines, adipokines, and growth factors, such as interleukin (IL)-6, insulin-like growth factor 1 (IGF-1), vascular endothelial growth factor (VEGF), tumor necrosis factor alpha (TNF-α), and stromal cell-derived factor 1 (SDF-1), which can initiate secondary genetic hits in genomically unstable MM clones, leading to myelomagenesis [8].

The primary cellular constituents of the BMME include mesenchymal stromal cells (MSCs), osteoblasts, osteoclasts, adipocytes, macrophages, and natural killer cells. Disruption of their functional balance shifts the BMME from a homeostatic state toward one that supports tumor progression [9]. MSCs in MM retain self-renewal capacity and can differentiate into osteoblasts, osteocytes, and adipocytes. Dysregulation of feedback loops between MSCs and MM cells results in altered levels of IL-7, transforming growth factor beta (TGF-β), and macrophage inflammatory protein-1 (MIP-1), promoting an immunosuppressive environment and suppressing pre-B cell development. Additionally, MM-MSC-derived microRNAs may inhibit osteoblastogenesis, thereby contributing to the imbalance in bone remodeling characteristic of MM [10].

Osteoblasts, responsible for bone mineralization, have an ambiguous role in MM. Some evidence indicates that osteoblast-derived decorin induces apoptosis and cell cycle arrest in MM cells, thereby inhibiting disease progression [11]. Conversely, other studies suggest that osteoblast-secreted factors like osteocalcin, osteopontin, TGF-β, and fibroblast growth factors (FGF) create a supportive niche for MM cells within the BMME [12].

Among bone-resorptive mechanisms, the receptor activator of nuclear factor-κB (RANK)/receptor activator of nuclear factor-κB ligand (RANKL)/osteoprotegerin (OPG) pathway is especially critical. RANK is expressed on osteoclasts, while RANKL is synthesized by MSCs, osteocytes, osteoblasts, and MM cells [13]. RANK-RANKL interactions accelerate osteoclast maturation, whereas OPG, produced by osteoblasts and MSCs, acts as a decoy receptor that inhibits RANKL binding. MM disrupts this regulatory system by downregulating OPG and increasing the RANKL/OPG ratio, fostering osteoclast activation and bone destruction [14].

Osteocytes, which account for the majority of bone cells (~95%), produce sclerostin under stress conditions. This protein inhibits MSC differentiation into osteoblasts by blocking Wnt signaling. Elevated levels of sclerostin, which is also secreted by MM cells, have been associated with increased disease severity [15].

Adipocytes, in addition to their metabolic role, secrete adipokines (e.g., leptin, adiponectin) and cytokines (e.g., IL-6, TNF-α, TGF-β), contributing to the energy and chemokine needs of proliferating MM cells [16]. This may help explain the increased MM incidence among obese individuals [17].

Syndecan-1 (CD138) is a heparan sulfate proteoglycan that mediates MM cell adherence to the BMME. It also contributes to bone pathology by reducing RANKL inhibitors, thereby increasing the RANKL/OPG ratio and promoting osteoclastogenesis [18,19].

Potential role of MOTS-c in MM and prognosis

MOTS-c is a peptide encoded by mitochondrial 12S rRNA and translated in the cytoplasm. It consists of 100-150 amino acids [20]. Under cellular stress conditions such as energy depletion and hypoxia, MOTS-c facilitates cellular adaptation by modulating folate metabolism, increasing intracellular 5-aminimidazole-4-carboxamide ribonucleotide (AICAR) levels, and activating 5’-monophosphate-activated protein kinase (AMPK). This signaling cascade influences glucose homeostasis, insulin sensitivity, inflammatory responses, exercise physiology, and aging [21].

Under glucose deprivation or hypoxia, MOTS-c translocates to the nucleus via AMPK signaling and promotes gene expression associated with anti-inflammatory cytokine release and cellular energy stabilization [22]. Due to these pleiotropic effects, MOTS-c is often considered functionally analogous to cytokines [23].

Studies have shown that AMPK signaling is pivotal for osteoblast proliferation, differentiation, and mineralization [24]. In an osteoporosis model, MOTS-c was found to promote osteogenesis of bone marrow-derived MSCs via the SMAD/TGF-β pathway, suggesting a protective role against bone loss [25].

In animal models of cancer-induced bone pain, administration of MOTS-c enhanced the OPG/RANKL ratio, downregulated RANK, RANKL, and TRAP expression, and thereby inhibited osteoclastogenesis and bone degradation [26].

Several scoring systems have been proposed to predict MM prognosis. Given the clinical heterogeneity of MM and the rapid evolution of therapeutic strategies, these systems are constantly evolving. Current models include the International Staging System (ISS) [27], Revised ISS (R-ISS) [28], Revised2 ISS (R2-ISS) [29], and Mayo Additive Score [30]. While ISS considers albumin and beta-2 microglobulin levels, R-ISS adds LDH and cytogenetic abnormalities (t(4;14), t(14;16), and del17p). R2-ISS omits t(14;16) and incorporates 1q amplification, reflecting recent data questioning the prognostic value of t(14;16) [31].

Despite advances in risk stratification, differences in progression-free survival (PFS) and overall survival (OS) persist among patients with similar scores, as highlighted by Alzahrani et al. in a study of R2-ISS stage 1-2 patients treated with quadruplet CD38-based regimens [32]. This points to the potential value of incorporating factors such as comorbidities, performance status, age, and bone disease burden. Considering the prominent skeletal pathology in MM, bone-related markers such as MOTS-c may offer valuable prognostic insights. In this context, our study explored the prognostic potential of MOTS-c by assessing its levels before and after treatment

## Materials and methods

Study design and population

This prospective cross-sectional study was conducted between January 2021 and December 2023 at the Hematology Department of Pamukkale University Hospital, Denizli, Türkiye. The study was approved by the Pamukkale University Non-Interventional Clinical Research Ethics Committee (approval number: E-60116787-020-189598).

Eligibility criteria

Inclusion criteria were age ≥18 years, newly diagnosed and treatment-naïve MM according to the International Myeloma Working Group (IMWG) diagnostic criteria [33], availability of complete baseline laboratory parameters, and the ability to provide informed consent. Patients with renal failure unrelated to MM (estimated glomerular filtration rate (eGFR) <30 mL/minute/1.73 m²), chronic inflammatory or autoimmune diseases, active infection, uncontrolled diabetes or hypertension, recent corticosteroid use, medications affecting metabolic or mitochondrial function (e.g., metformin, statins), and prior chemotherapy or radiotherapy were excluded. No healthy control group was included due to the prospective design of the study.

A total of 29 patients with newly diagnosed MM were enrolled; 23 were classified as responders and six as refractory based on treatment outcomes.

Laboratory assessments

Baseline and post-treatment measurements included serum immunoglobulins (IgG, IgA, IgM), creatinine, eGFR, electrolytes, liver enzymes, total protein, albumin, beta-2 microglobulin, alkaline phosphatase (ALP), lactate dehydrogenase (LDH), calcium, WBC, neutrophils, hemoglobin, and platelets. Routine biochemical analyses were performed using a Cobas 702 analyzer (Roche Holding AG, Basel, Switzerland). GFR was estimated using the creatinine-based eGFRcr equation [34]:

\(\text{eGFR} = 141 \times \min\left(\frac{\text{Scr}}{\kappa}, 1\right)^{\alpha} \times \max\left(\frac{\text{Scr}}{\kappa}, 1\right)^{-1.209} \times 0.993^{\text{Age}} \times [1.018 \text{ if female}] \times [1.159 \text{ if Black}]\), where Scr: serum creatinine (mg/dL), κ = 0.7 (female), 0.9 (male), α = −0.329 (female), −0.411 (male) 

Fasting venous blood samples were collected between 08:00 and 10:00 AM to minimize circadian variation. Serum tubes were allowed to clot for 30 minutes at room temperature and were then centrifuged at 3,000 rpm for 10 minutes. The resulting serum supernatant was aliquoted into 0.5 mL polypropylene tubes to prevent multiple freeze-thaw cycles. All samples were stored at -80°C until analysis, and MOTS-c measurements were performed within six months of sample collection.

Serum MOTS-c concentrations were measured using a commercially available ELISA kit (BT Lab, Shanghai, China). The assay detection range was 5-200 ng/mL, with an analytical sensitivity of 1.8 ng/mL. The intra-assay coefficient of variation was <8%, and the inter-assay coefficient of variation was <10%, as reported by the manufacturer. All samples were measured in duplicate, and mean values were used for analysis. Calibration curves were constructed according to the kit protocol.

Statistical analysis

Data normality was assessed using the Kolmogorov-Smirnov test. As most variables, including MOTS-c levels, showed a non-normal distribution and the sample size was relatively small, non-parametric statistical tests were selected. Continuous variables were summarized as medians with interquartile ranges (25th-75th percentiles), and categorical variables as frequencies and percentages. Group comparisons were performed using the Wilcoxon signed-rank test for paired analyses, and the Mann-Whitney U or Kruskal-Wallis tests for independent group comparisons, as appropriate. Receiver operating characteristic (ROC) curves were generated to evaluate the discriminative ability of MOTS-c. No correction for multiple comparisons was applied due to the exploratory nature of the study. All analyses were two-sided with a significance threshold of p < 0.05 and were conducted using SPSS Statistics for Windows, version 15.0 (SPSS Inc., Chicago, Illinois, United States).

## Results

The cohort included 15 males (51.7%) and 14 females (48.3%). The responder group comprised 23 patients (79.3%), and the refractory group comprised six patients (20.7%). No statistically significant differences in age or sex distribution were observed between groups (p > 0.05).

In patients who responded to treatment, MOTS-c concentrations showed a clear upward trend, rising from a baseline median of 44.88 ng/mL to 54.87 ng/mL, with a wider interquartile range suggesting increased variability that may be linked to metabolic adaptation. By contrast, refractory patients exhibited almost unchanged levels (32.16 → 36.51 ng/mL), with post-treatment values remaining narrowly distributed, indicative of a blunted or absent activation of mitochondrial-linked signaling. Consistent with these patterns, baseline MOTS-c values were numerically higher in responders (44.88 ± 35.05 ng/mL) compared with refractory patients (32.16 ± 14.91 ng/mL), although this difference did not reach statistical significance. Following therapy, MOTS-c levels increased to 54.87 ± 19.34 ng/mL in responders and to 36.51 ± 5.43 ng/mL in refractory cases, with no significant within-group changes (p = 0.2208 and p = 0.4321, respectively). The mean ΔMOTS-c was greater in responders (10.00 ± 38.05 ng/mL) compared to refractory patients (4.35 ± 12.48 ng/mL), reflecting higher inter-individual variability in those who achieved a clinical response (Table 1).

**Table 1:** MOTS-c level comparison between responders and refractory patients. Comparisons between pre- and post-treatment values were performed using the Wilcoxon signed-rank test. A two-sided p < 0.05 was considered statistically significant. MOTS-c: mitochondrial open reading frame of the 12S rRNA-c

Group	Baseline MOTS-c, mean ± SD	Post-treatment MOTS-c, mean ± SD	Δ MOTS-c, mean ± SD	p-value
Responders	44.88 ± 35.05	54.87 ± 19.34	10.00 ± 38.05	0.2208
Refractory	32.16 ± 14.91	36.51 ± 5.43	4.35 ± 12.48	0.4321

To explore whether baseline MOTS-c levels could differentiate between treatment-responsive and refractory multiple myeloma cases, a non-parametric comparison was conducted. Specifically, the Mann-Whitney U test was applied to pre-treatment MOTS-c concentrations from both patient subsets. The analysis revealed no statistically significant divergence in distributions (U = 76.0, p = 0.726), suggesting that initial mitochondrial-derived peptide levels may not independently predict early therapeutic response. These findings imply a limited discriminative capacity of MOTS-c at diagnosis for forecasting resistance to initial treatment regimens.

**Figure 1:**
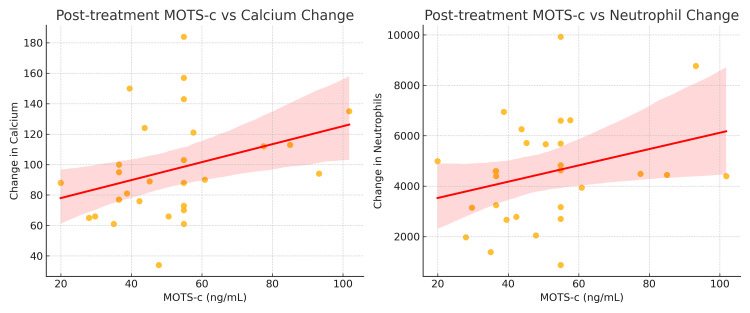
Inverse association between post-treatment MOTS-c levels and biochemical shifts in calcium and neutrophil counts. Scatter plots demonstrate correlations between post-treatment MOTS-c concentrations and changes in (A) serum calcium and (B) neutrophil counts. Correlation analyses were performed using Spearman’s rank correlation test. Shaded areas represent 95% confidence intervals of the regression line. A two-sided p < 0.05 was considered statistically significant. MOTS-c: mitochondrial open reading frame of the 12S rRNA-c

In addition to group-level comparisons, correlation analyses were performed to investigate the relationship between post-treatment MOTS-c levels and dynamic changes in selected biochemical markers. MOTS-c levels at the end of treatment demonstrated a moderate negative association with Δ calcium values, suggesting that greater reductions in calcium were observed in patients with elevated MOTS-c levels post-therapy (Figure 1). This pattern may reflect the potential influence of MOTS-c on bone resorption and remodeling, consistent with its reported modulatory role on the RANKL/OPG axis. Conversely, Δ neutrophil counts showed a dispersed, non-directional relationship with MOTS-c, implying that post-treatment neutrophil variability may not be tightly linked to mitochondrial signaling or inflammatory resolution mediated by MOTS-c. These findings support the hypothesis that MOTS-c is more closely involved in metabolic and skeletal homeostasis than in direct modulation of peripheral leukocyte profiles.

When examining MOTS-c shifts after treatment across various clinical subgroups, some interesting patterns began to emerge (Table 2). Although the difference between men and women was relatively small, individuals under the age of 60 tended to show a noticeably greater increase in MOTS-c levels than their older counterparts. In terms of disease subtype, patients with IgA lambda myeloma had the most pronounced change, hinting at possible metabolic sensitivity in this group. The contrast between responsive and refractory cases was especially telling-responders exhibited a considerable rise in MOTS-c following therapy, whereas those in the refractory group showed barely any change. These trends may suggest that MOTS-c shifts reflect not only general metabolic activity, but also the biological readiness of certain patients to respond more favorably to treatment (Table 2).

**Table 2:** Analysis of MOTS-c levels according to clinical subgroups. Data are expressed as n (%) and median (IQR, 25th–75th percentile). Comparisons between groups were performed using the Mann–Whitney U test (for two-group comparisons) or the Kruskal–Wallis test (for comparisons among more than two groups). A two-sided p < 0.05 was considered statistically significant. Ig, immunoglobulin; MM, multiple myeloma; MOTS-c, mitochondrial open reading frame of the 12S rRNA-c

Subgroups, n (%)			MOTS-c at diagnosis, median (IQR)	Post-treatment MOTS-c level, median (IQR)	Δ MOTS-c level, median (IQR)
Gender	male	15 (51%)	32.16 (25.80-41.95	45.15 (36.51-57.60)	13.41 (1.76-23.26)
	female	14 (49%)	32.08 (30-53.50)	52.72 (36.51-54.87)	15.15 (-16.98-24.87)
p			0.715	0.813	0.715
Age	<60 years	17 (58.6%)	32.00 (27.00-36.64	54.87 (39.48-54.87)	16.77 (4.35-24.87)
	≥60 years	12 (41%)	39.48 (25.40-48.44)	43.70 (35.77-56.23)	2.83 (-15.00-20.94)
p			0.556	0.499	0.227
MM Type	Ig A Kappa	4 (13.8%)	23.32 (15.99-46.84)	46.80 (34.21-54.87)	10.90 (-4.56-30.81)
	Ig G Kappa	11 (37.9%)	36.52 (30.00-54.27)	54.87 (36.51-57.60)	12.41 (-5.21-24.87)
	Ig A Lambda	2 (6.9%)	35.98 (30.00-41.95)	57.86 (54.87-60.85)	21.88 (12.92-30.85)
	Ig G Lambda	2 (6.9%)	42.75 (32.00-53.50)	45.70 (36.51-54.87)	2.94 (-16.99-22.87)
p			0.768	0.502	0.904
Light Chain	Kappa	4 (13.8%)	32.16 (20.83-54.27)	47.80 (36.51-54.87)	12.41 (-5.21-23.26)
	Lambda	6 (20%)	34.32 (30.00-41.95)	54.87 (43.77-60.85)	17.50 (1.76-30.85)
p			0.668	0.308	0.403
Refractory	Yes	6 (20.7%)	32.16 (16.21-43.39)	36.51 (36.44-36.51)	4.35 (1.76-12.41)
	No	23 (79.3%)	32.00 (26.07-41.95)	54.87 (42.26-57.60)	17.89 (-5.21-24.87)
p			0.733	0.006	0.302

**Figure 2:**
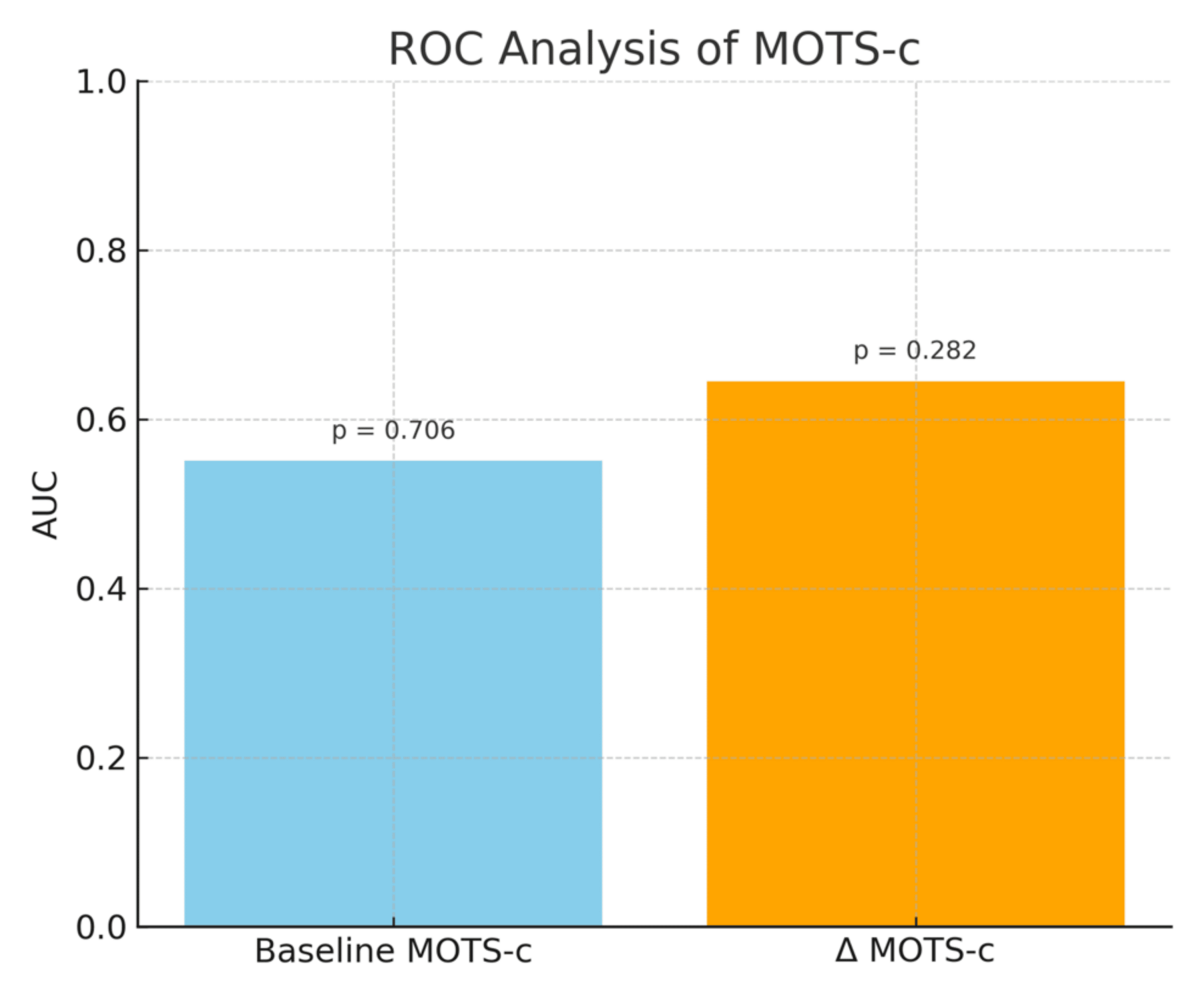
ROC analysis of MOTS-c levels in predicting treatment response Receiver operating characteristic (ROC) curves were used to evaluate the discriminative ability of baseline and Δ (post–pre) MOTS-c levels for distinguishing responders from refractory patients. Analyses were performed using the ROC curve method, and the area under the curve (AUC) values are presented with their corresponding p-values. A two-sided p < 0.05 was considered statistically significant. MOTS-c: mitochondrial open reading frame of the 12S rRNA-c

ROC analysis yielded limited prognostic value for baseline (area under the curve (AUC) = 0.551, p = 0.706) and Δ MOTS-c (AUC = 0.645, p = 0.282) (Figure 2).

## Discussion

**Figure 3:**
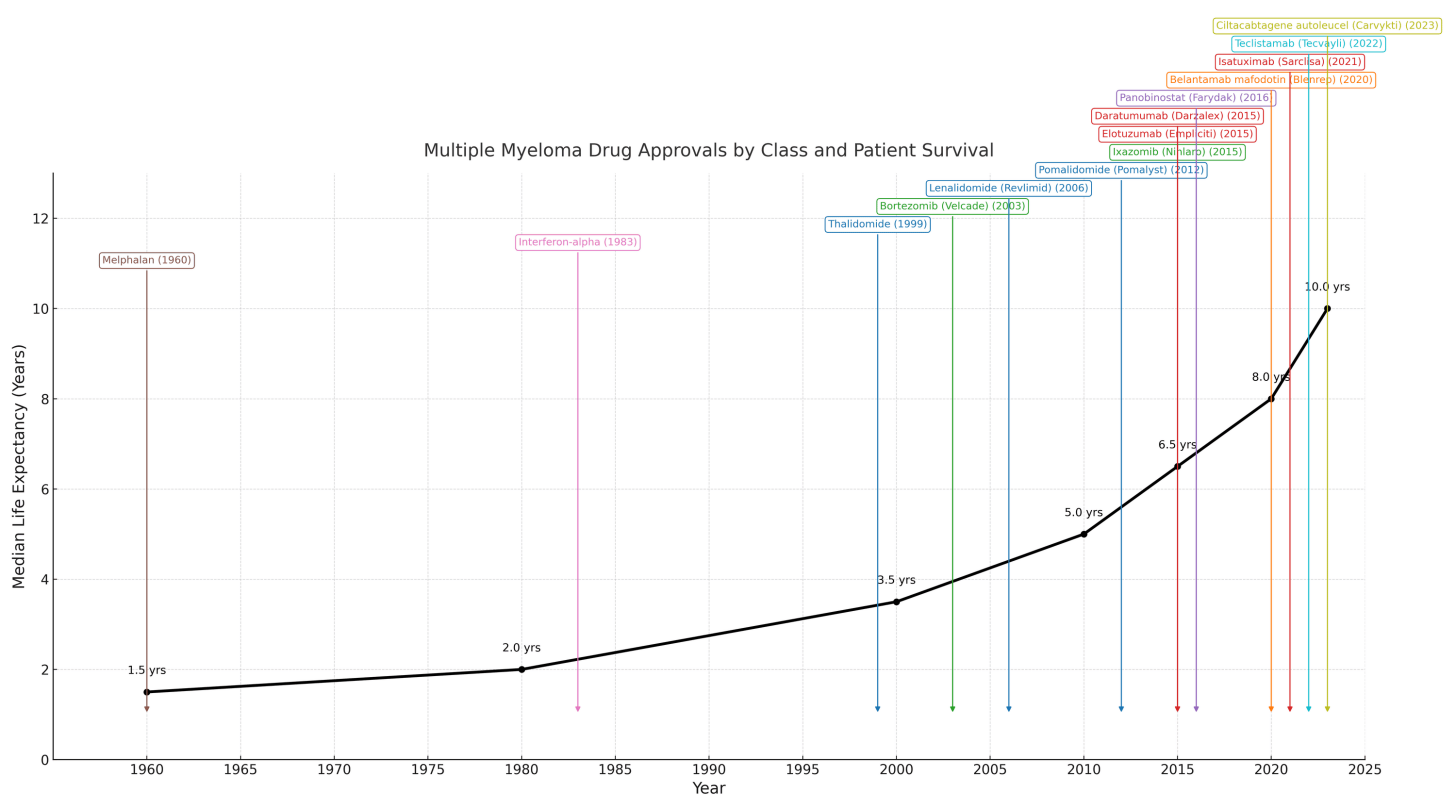
Multiple myeloma drugs approval by class and patient survival

Over the last two decades, the incorporation of proteasome inhibitors, immunomodulatory agents, CD38-targeted antibodies, and BCMA-directed therapies into frontline and relapse regimens has markedly improved survival outcomes in MM, particularly when used in multidrug combinations, as summarized in Figure 3. Given the rapidly evolving therapeutic landscape of MM, driven by shifting biological targets and increasingly potent pharmacologic strategies, there is a growing need to refine and update prognostic markers so that they better capture treatment-related biological dynamics. 

This investigation examined the relationship between serum MOTS-c levels and therapeutic outcomes in individuals newly diagnosed with MM, focusing particularly on elevation in MOTS-c among patients who responded to therapy, whereas such changes were absent in refractory cases (Table 2). Although baseline levels of MOTS-c did not show a direct correlation with conventional prognostic indices, the notably diminished levels following therapy in refractory patients imply a potential association with resistance mechanisms in MM.

**Figure 4:**
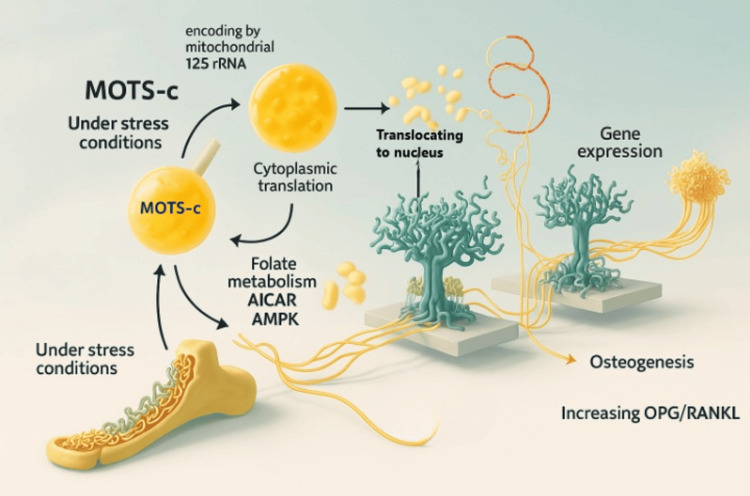
Schematic representation of the stress-induced mitochondrial peptide MOTS-c and its downstream signaling effects MOTS-c, mitochondrial open reading frame of the 12S rRNA-c; AICAR, 5-aminimidazole-4-carboxamide ribonucleotide; AMPK, activating 5’-monophosphate-activated protein kinase; OPG, osteoprotegerin; RANKL, receptor activator of nuclear factor-κB ligand Image Credit: Authors

The observed elevation in MOTS-c post-intervention among responders (p = 0.0004) underscores its potential involvement in immunometabolic regulation within the marrow environment. This peptide is known to influence cellular energy regulation and apoptotic control by activating AMPK pathways and mediating anti-inflammatory cytokine expression during metabolic stress [20-22]. Given AMPK’s established role in osteoblast function and bone remodeling [24], MOTS-c may act on both immunologic and skeletal fronts in MM. MOTS-c, as a stress-responsive mitochondrial peptide, modulates AMPK-mediated metabolic adaptation, anti-inflammatory signaling, and bone-remodeling pathways, as illustrated in Figure 4.

Experimental studies have shown that MOTS-c modulates the RANKL/OPG balance, suppressing osteoclast activity and reducing bone degradation [25]. Considering the pivotal role of the bone marrow milieu, particularly the dysregulation in osteoclast-osteoblast dynamics mediated by RANK/RANKL/OPG signaling [14,18]. MOTS-c could indirectly influence disease containment via microenvironmental modulation.

Exploratory subgroup analyses highlighted a near-significant association between MOTS-c levels and immunoglobulin subtypes, although this was not statistically confirmed in patients with IgG-secreting disease. Notably, in the refractory group, strong correlations (r > 0.9) between MOTS-c changes and IgG variations were observed, though they did not achieve significance, likely due to limited sample size (Table 2). These trends point to a possible attenuation of humoral and metabolic responsiveness in treatment-resistant individuals.

Subgroup analyses provided additional insights. Younger patients (<60 years) showed greater post-treatment increases in MOTS-c levels compared to older individuals, which may be attributable to better mitochondrial plasticity and reduced metabolic exhaustion in younger marrow environments. Similarly, patients with IgA lambda myeloma exhibited the most pronounced MOTS-c elevation, suggesting potential subtype-specific metabolic vulnerabilities that could be further investigated (Table 3).

**Table 3:** Comparison of parameters between groups before and after treatment. Data are expressed as median (IQR, 25th–75th percentile). Intra-group comparisons of pre- and post-treatment values were performed using the Wilcoxon signed-rank test, while inter-group comparisons between responders and refractory patients were analyzed using the Mann–Whitney U test. A two-sided p < 0.05 was considered statistically significant. IgG, immunoglobulin G; IgM, immunoglobulin M; IgA, immunoglobulin A; GFR, glomerular filtration rate; β₂-microglobulin, beta-2 microglobulin; LDH, lactate dehydrogenase; WBC, white blood cell; m, male; f, female.

Parameters	Overall (n=29, 100%)			Refractory (n=6, 23%)			Responsive (n=23, 77%)		
	Before Treatment, median (IQR)	After Treatment, median (IQR)	p-value	Before Treatment, median (IQR)	After Treatment, median (IQR)	p-value	Before Treatment, median (IQR)	After Treatment, median (IQR)	p-value
IgG (g/L)	11.00 (6.58–29.00)	6.88 (4.65–9.62)	<0.001	20.50 (13.00–41.50)	10.61 (7.95–11.70)	0.028	9.08 (5.90–29.00)	6.88 (4.15–7.26)	0.006
IgM (g/L)	0.35 (0.18–0.56)	0.33 (0.17–0.39)	0.381	0.40 (0.19–0.60)	0.37 (0.12–0.39)	0.400	0.35 (0.15–0.56)	0.33 (0.17–0.39)	0.594
IgA (g/L)	0.73 (0.27–1.55)	0.81 (0.18–0.91)	0.011	0.55 (0.26–1.12)	0.17 (0.12–0.91)	0.027	0.73 (0.34–16.45)	0.87 (0.22–1.09)	0.031
Creatinine (mg/dL)	0.94 (0.71–1.69)	0.89 (0.59–1.03)	0.029	0.88 (0.72–0.94)	0.69 (0.57–1.00)	0.173	0.94 (0.70–1.71)	0.94 (0.62–1.03)	0.086
GFR (mL/min/1.73 m²)	86 (43–99)	85 (78–103)	0.012	91 (76–93)	93 (78–103)	0.345	79 (35–103)	85 (77–107)	0.018
Total Protein (g/L)	78.3 (70.0–90.0)	62.3 (61.0–65.9)	<0.001	76.0 (70.0–94.0)	68.35 (63.3–75.4)	0.116	78.3 (69.4–90.0)	62.30 (59.6–64.86)	<0.001
Albumin (g/L)	39.7 (34.0–42.9)	41.2 (40.0–44.3)	0.015	40.5 (34.0–48.0)	41.45 (38.8–47.0)	0.463	39.7 (32.8–42.9)	41.20 (40.00–44.30)	0.024
β2-Microglobulin (mg/L)	4.49 (2.71–7.12)	2.82 (2.35–5.04)	0.076	2.57 (1.99–7.62)	2.82 (2.35–3.55)	0.833	4.57 (2.79–7.12)	2.86 (2.34–5.04)	0.083
LDH (U/L)	184 (160–220)	163 (136–183)	0.019	199 (134–281)	160 (142–206)	0.600	183 (160–220)	163 (135–183)	0.020
Calcium (mg/dL)	9.51 (9.20–10.29)	9.00 (8.85–9.27)	<0.001	9.19 (9.17–9.46)	9.06 (8.55–9.51)	0.345	9.63 (9.26–11.00)	9.00 (8.90–9.21)	0.001
WBC (×10³/μL)	7700 (5660–8900)	4710 (4130–5360)	<0.001	7555 (5660–7750)	5020 (3290–5870)	0.046	7700 (5640–9390)	4710 (4130–5080)	<0.001
Neutrophil (×10³/μL)	4490 (3150–5690)	2820 (2020–3160)	<0.001	4500 (3250–4600)	2990 (2490–3540)	0.116	4490 (2780–6260)	2820 (1890–3030)	0.002
Hemoglobin (g/dL)	10.6 (8.5–11.8)	11.6 (11.1–12.3)	0.119	11.0 (9.6–16.3)	12.4 (8.8–13.5)	0.753	10.6 (8.4–11.8)	11.6 (11.1–12.0)	0.030
Platelet (×10³/μL)	244 (195–307)	218 (177–243)	0.147	222 (195–300)	189 (154–225)	0.173	244 (186–326)	218 (186–249)	0.330
MOTS-c (ng/mL)	32.160 (26.07–41.95)	50.57 (36.51–54.87)	0.037	32.16 (16.21–43.39)	36.51 (36.44–36.51)	0.248	32.00 (26.07–41.95)	54.87 (42.26–57.60)	0.068

ROC analysis failed to identify strong predictive capacity for MOTS-c in either baseline or delta measurements (AUC: 0.551 and 0.645, respectively; p > 0.05). However, the persistently reduced levels in the non-responsive group suggest that MOTS-c signaling may be disrupted in refractory disease states. In light of MM's biological diversity, current risk stratification tools-ISS, R-ISS, and R2-ISS-may benefit from enhancement through inclusion of metabolic or immunologic indicators such as MOTS-c [31,32].

While the findings presented here offer valuable insights into the potential role of MOTS-c in multiple myeloma, several strengths and limitations merit emphasis. A major strength of this study is its paired assessment of MOTS-c alongside a broad panel of biochemical markers before and after treatment, allowing for intra-individual evaluation of metabolic shifts rather than relying solely on cross-sectional comparisons. By focusing on a relatively novel mitochondrial-derived peptide with emerging immunometabolic relevance, the study contributes exploratory data to an underdeveloped area of myeloma biomarker research.

However, important limitations must also be acknowledged. The relatively small cohort size, particularly the limited number of refractory patients, reduced statistical power and restricted the ability to perform multivariate analyses, potentially explaining borderline or non-significant associations. The absence of longitudinal endpoints, such as progression-free or overall survival, further limits conclusions regarding the prognostic implications of MOTS-c. Variation in treatment regimens and the inability to fully account for potential confounders, including diet, physical activity, concurrent medications, and metabolic comorbidities, may have influenced circulating MOTS-c levels and introduced uncertainty regarding internal validity. These factors also limit the generalizability of the findings to broader MM populations. Therefore, although the observed trends are biologically plausible and hypothesis-generating, the results should be interpreted with appropriate caution. Larger, prospective studies with standardized treatments and extended follow-up are needed to validate the clinical significance of MOTS-c in MM.

## Conclusions

Serum MOTS-c levels demonstrated an upward trend in patients who responded to therapy, suggesting a potential association between treatment effectiveness and MOTS-c dynamics. However, given the small sample size and observational design, these findings should be regarded as preliminary. MOTS-c does not appear to function as an independent prognostic marker in its current form, but it may hold value as an exploratory adjunct for monitoring metabolic responses during treatment. Larger, prospective studies with standardized treatment protocols and longitudinal follow-up are needed to clarify its prognostic relevance and to determine whether MOTS-c could eventually be integrated into future risk-stratification frameworks in multiple myeloma.
